# ENDOCRINOLOGY IN THE TIME OF COVID-19: Management of calcium metabolic disorders and osteoporosisThis manuscript is part of a commissioned series of urgent clinical guidance documents on the management of endocrine conditions in the time of COVID-19. This clinical guidance document underwent expedited open peer review by Bente Langdahl (Aarhus University, Denmark), Karin Amrein (Medical Univesity of Graz, Austria), and Maria Lousa Brandi (University of Florence, Italy)

**DOI:** 10.1530/EJE-20-0385

**Published:** 2020-08-01

**Authors:** Neil J Gittoes, Sherwin Criseno, Natasha M Appelman-Dijkstra, Jens Bollerslev, Ernesto Canalis, Lars Rejnmark, Zaki Hassan-Smith

**Affiliations:** 1 Department of Endocrinology, Queen Elizabeth Hospital Birmingham, University Hospitals Birmingham NHS Foundation Trust, Birmingham, UK; 2 Centre for Endocrinology, Diabetes and Metabolism, Birmingham Health Partners, Birmingham, UK; 3 Institute of Metabolism and Systems Research, College of Medical and Dental Sciences, University of Birmingham, Birmingham, UK; 4 Centre for Bone Quality, Leiden University Medical Centre, Leiden, The Netherlands; 5 Section of Specialized Endocrinology, Oslo University Hospital, Oslo, Norway; 6 Faculty of Medicine, University of Oslo, Oslo, Norway; 7 Departments of Orthopaedics and Medicine, UConn Health, Farmington, Connecticut, USA; 8 Department of Endocrinology and Internal Medicine, Aarhus University Hospital, Aarhus, Denmark

## Abstract

Endocrinologists have had to make rapid changes to services so that resources can be focused on the COVID-19 response to help prevent spread of the virus. Herein we provide pragmatic advice on the management of commonly encountered calcium metabolic problems and osteoporosis. Non-urgent elective appointments should be postponed, and remote consultations and digital health solutions promoted. Patients should be empowered to self-manage their conditions safely. Patients, their caregivers and healthcare providers should be directed to assured national or international online resources and specific patient groups. For patients in acute hospital settings, existing emergency guidance on the management of hyper- and hypo-calcaemia should be followed. An approach to osteoporosis management is outlined. IV zoledronic acid infusions can be delayed for 6–9 months during the pandemic. Patients established on denosumab, teriparatide and abaloparatide should continue planned therapy. In the event of supply issues with teriparatide or abaloparatide, pausing this treatment in the short term is likely to be relatively harmless, whereas delaying denosumab may cause an immediate increased risk of fracture. The challenge of this pandemic will act as a catalyst to innovate within our management of metabolic bone and mineral disorders to ensure best use of resources and resilience of healthcare systems in its aftermath.

## Introduction

The emergence of the 2019 novel coronavirus (COVID-19) in Wuhan, China and subsequent global spread has placed health systems under unprecedented strain (1). Endocrinologists, amongst others, have had to focus on contribution to acute care in this initial phase. There are a number of considerations as to how we respond as endocrine and metabolic bone disease specialists. The first, concerns how we ensure that we have effective mechanisms in place to postpone non-urgent elective activity whilst ensuring that we do no harm. As we have seen this has involved adopting new ways of working such as remote consultations in line with European Society for Endocrinology (ESE) guidance (https://www.ese-hormones.org/media/2223/covid-and-endocrine-diseases-ese-statement-final_23032020.pdf). These measures are required to maintain resilience in our health care systems and to contribute to effective physical distancing to prevent disease spread. Clear communication with effective messaging and educational support are central to this.

Many centres have set up helplines run by clinical specialist nurses supported by consultants. IT systems can be used to provide remote ‘advice and guidance’ to GPs and non-specialist colleagues. Patients should be directed to online resources and helplines set-up by specialist societies, organisations and patient groups to provide support during the pandemic (Table 1).

**Table 1:** Summary of online resources available to support management of calcium and metabolic bone conditions during the COVID-19 pandemic.

Society for Endocrinology
• COVID-19 resources for managing endocrine conditions
• www.endocrinology.org/clinical-practice/covid-19-resources-for-managing-endocrine-conditions/
• Resources related to metabolic bone conditions include: ⚬ Letter templates for denosumab during the COVID-19 pandemic ⚬ Denosumab patient information leaflet ⚬ Letter template for postponement of zoledronic acid infusion ⚬ COVID-19 and parathyroid condition FAQs
Royal Osteoporosis Society
• Free osteoporosis patient helpline +44 800 800 0035
• Includes large resource of practical advice and information for patients and carers
• Advice and guidance for health care professionals on managing osteoporosis through COVID-19 response
• https://theros.org.uk/information-and-support/coronavirus-and-osteoporosis
International Osteoporosis Foundation
• Questions and answers for patients, caregivers and healthcare providers
• Links to other relevant international societies
• https://www.iofbonehealth.org/news/covid-19-and-osteoporosis
National Osteoporosis Foundation
Offers guidelines through a recorded webinar by a group of experts http://www.nof.org/covid-19-updates/
European Reference Network on Rare Bone Diseases COVID-19 page (https://ernbond.eu/covid-19-emergency/)
• Includes links to advice from international bodies on the management of rare bone diseases and the impact of the pandemic (http://ernbond.eu/indications/)
Parathyroid UK
• https://parathyroiduk.org/news/coronavirus-advice-for-people-with-hypoparathyroidism/
European Society of Endocrinology (ESE) COVID-19 resources
• https://www.ese-hormones.org/about-us/our-communities/clinicians/covid-19-and-endocrine-disease-clinical-information-and-comment-from-ese/
ESE information leaflet on the treatment of chronic hypoparathyroidism in adults
• https://www.ese-ormones.org/media/1353/hypoparathyroidismpatientleaflet.pdf
Endocrine Society
As of this writing, the Endocrine Society is developing resources for clinicians managing endocrine problems during the pandemic
www.endocrine.org/

We must ensure that we identify patients with complex health needs and provide effective management strategies during this time. We need to be mindful of planning over the medium term to prevent excess chronic disease morbidity. The aftermath to the pandemic will not likely be characterised by a return to business as usual. We will need to take what we have learned during this acute phase to develop novel models of working. This will involve careful consideration of where we add value as specialists. It is clear that we will need to harness multi-disciplinary working and digital health solutions to meet this challenge.

The management approach to calcium metabolic disorders needs to be tailored to the setting, such as in large >1000 bed ‘field hospitals’, where close monitoring of fluid balance and biochemical tests may not be possible (2). In this review we focus on the impact of COVID-19 on the management of calcium metabolic and bone conditions focusing on hypo- and hyper-calcaemia and osteoporosis.

## Are patients with calcium disorders and osteoporosis at increased risk from COVID-19?

- No. Mineral and metabolic bone conditions may be an added complication in patients presenting with COVID-19, although at present there is **no evidence** to suggest that patients with these conditions are at increased risk from the infection. Observational studies have hypothesised an increased risk of hospitalization due to infections in patients with hypoparathyroidism. It is, however, unknown whether a causal relationship exists, and no data are available on risk of COVID-19 infections in hypoparathyroidism.
- It has been proposed that vitamin D may play a role in reducing the risk of respiratory infections, such as seasonal influenza, via modulation of inflammatory cytokine profiles and induction of cathelicidins and defensins (3, 4). Previous observational research has identified **vitamin D deficiency as a risk factor for ARDS and severity of ARDS** (5), which can be a life-threatening complication of COVID-19. There is heterogeneity in outcomes and confounding factors in previous observational and RCT studies in this area.
- A systematic review and meta-analysis of the use of vitamin D supplementation to prevent acute respiratory infections concluded that the intervention was safe and efficacious overall, with a low number needed to treat (4), particularly in those with severe vitamin D deficiency and with daily/weekly dosing. **At the time of writing no reliable data on this issue specific to COVID-19 are available.**
- Ensuring that vitamin D status is optimised to ‘sufficient’ levels is a core component of the management of metabolic bone diseases, so it is important that loading or maintenance supplementation is administered as appropriate.

## How will COVID-19 impact on patients with calcium metabolic disorders and osteoporosis?

- The majority of metabolic bone and mineral diseases have a chronic natural history and are managed in an outpatient setting. The inability to run face-to-face outpatient appointments, promotes the use of remote follow-up such as telephone or video consultations.
- Once a cause has been elucidated for disturbances in mineral metabolism, management is usually driven by regular review and blood test monitoring. Patients shielding or self-isolating will not have access to regular blood test monitoring and decisions will need to be made as to whether to lengthen **intervals between blood tests** to inform clinical decision-making.
- Redeployment of outpatient-based staff to inpatient COVID-19 areas means that there is significantly reduced workforce to deliver outpatient services. **Helplines and online resources** can help bridge some of this gap in patient contacts.
- Disturbances of mineral metabolism can present symptomatically and acutely. Such problems need to be addressed conventionally as patients attend emergency departments (EDs). These patients should highlight their chronic disease to staff when entering the ED.
- Patients awaiting diagnostics such as DXA scanning and other imaging will and should be postponed, and in the majority of cases, this **will not impact negatively** upon care.
- Patients awaiting surgery such as **parathyroidectomy (PTX)** will and should be delayed in accessing definitive surgery.
- Patients receiving **parenteral forms of therapy for osteoporosis** administered by health providers are likely to experience significant hurdles to contend with to continue uninterrupted treatment.

## How to manage acutely unwell patients with hypercalcaemia or hypocalcaemia without full investigations

- Consideration needs to be made with regards to management of hyper- and hypo-calcaemia, in particular whether the healthcare facility is able to administer IV therapies if required, and whether in the event of these complications, transfer to an acute hospital would be appropriate or whether a palliative route may be more appropriate.
- The above decisions should be made ‘**at the front door**’ with input from senior decision makers such as general physicians, with involvement and support of disease specialists where available. During the pandemic we have found that proactive decision making, including prescription of anticipatory medications and involvement of palliative medicine teams to be extremely helpful.
- Wherever possible, **existing guidelines** should be followed.
- There is a paucity of guidance on the management of endocrine conditions for those patients who are at the end of life, however, and in general, we would advocate adhering to the principles of good palliative care in other chronic diseases including **avoiding unnecessary monitoring and investigations** while supporting discontinuation of medication that does not provide symptom relief. Intensive monitoring of fluid balance and routine blood tests are not desirable in patients with disturbances of mineral metabolism who are in the last days of life.

### Acute management of hypercalcaemia

- Irrespective of COVID-19 status, patients will continue to present with acute hypercalcaemia as defined by basic biochemical testing. The Society for Endocrinology **guideline on emergency management of acute hypercalcaemia** in adult patients provides a framework for use in acute hospital settings where basic lab results are available and should be followed in so far as feasible (6). If by any means possible, a diagnosis of hypercalcaemia should be followed by measurement of PTH levels, as this may help determine the necessity of immediate subsequent examinations/treatments.
- If serum PTH levels are high-normal or elevated, a diagnosis of primary hyperparathyroidism (PHPT) is most likely. **No further immediate examinations or treatment** are required unless a patient is severely symptomatic and/or serum calcium levels are >~3.25 mmol/L.
- Suppressed serum PTH levels may indicate a state of disequilibrium hypercalcemia due to for example, malignancies or vitamin D intoxication, which may worsen rapidly and necessitate immediate treatment.
- Standard acute management of severe hypercalcaemia involves rehydration with appropriate volumes of i.v. 0.9% saline. Response should be carefully monitored, and clinicians should be mindful that in COVID-19, there is a risk of **acute respiratory distress syndrome (ARDS) and this may be substantially worsened by overly liberal fluid management**. Treatment with calcitonin may also be helpful during the first 24 or 48 h in management of acute hypercalcaemia. It is important to be aware of the risk of fluid overload in older patients or those with renal impairment, which can be a manifestation of COVID-19. Careful monitoring is required. Small doses of IV furosemide may be considered in such scenarios but furosemide should only be used to manage fluid overload and not as an integral approach to managing the hypercalcaemia.
- If adverse clinical signs/symptoms continue and further treatment is needed, the approach depends on whether the hypercalcemia is considered to be caused by excess PTH or is of non-parathyroid origin (if feasible to have been elucidated). Approaches to treatment of PHPT are detailed below. If further treatment of non-parathyroid hypercalcemia (e.g. hypercalcemia of malignancy) is deemed necessary, IV zoledronic acid may be considered. Teams should be aware of **flu-like symptoms in the days following administration, a common side effect of this treatment**, however. If serum calcium levels are not reduced in response to bisphosphonate treatment, subcutaneous denosumab may be considered (approved for treatment of hypercalcaemia of malignancy refractory to bisphosphonate therapy in the USA), as well as treatment with glucocorticoids may be helpful in some cases.
- There is a risk of hypocalcaemia after IV bisphosphonates and S/C denosumab, even in those presenting with initial hypercalcaemia, particularly in those with **vitamin D deficiency**. Vitamin D supplementation should be pragmatically administered (~25,000–50,000 IU orally) if vitamin D deficiency is detected or there is concern about vitamin D deficiency and rebound hypocalcaemia.

### Acute management of hypocalcaemia

- As for hypercalcaemia, irrespective of COVID-19 status, patients will continue to present with acute hypocalcaemia that is identified by routine biochemistry testing. Acutely presenting hypocalcaemia is a **potentially life-threatening** emergency and requires hospital admission with appropriate monitoring and support.
- **Hypocalcaemia is often encountered in patients with infections and severe illness**. PTH response to hypocalcaemia due to infections is often blunted but variable. No data are available whether COVID-19 is associated with development of hypocalcaemia. Most patients with hypocalcaemia due to infections are asymptomatic, and there are no data supporting beneficial effects of treatment of asymptomatic patients without signs of hypocalcaemia caused by infections.
- In patients with symptomatic hypocalcaemia that is refractory to treatment with drug therapies issued and administered in a community setting, we advocate following the Society for Endocrinology **emergency guidance for acute hypocalcaemia** (7).
- Hypoparathyroid patients may encounter more unstable mineral metabolism during a COVID-19 infection.

## How should endocrine services for patients with calcium metabolic disorders and osteoporosis be remodelled in the acute crisis?

- **Promotion of effective self-management strategies** is essential for patients with chronic illnesses in the midst of the pandemic. The ‘4E’ framework (Educate, Equip, Engage, Empower) can be used to support this. This enables individuals to monitor their condition and to facilitate the cognitive, behavioural, and emotional responses necessary to maintain a satisfactory quality of life.
- The vast majority of patients with chronic metabolic bone and calcium disorders can have follow-up safely converted to remote review. Use of telephone calls or video consultations is appropriate in these patients. Routine letters to inform patients of changes in their management and follow-up plans should be sent, including who to contact in the event of any concerns with their condition.
- The need for routine blood tests should be reviewed and arrangements should be made for **blood testing away from the acute hospital sites** with ‘drive-through’, ‘pop-up’, GP and domiciliary phlebotomy services being suggested models.
- Many centres have set up **helplines** run by specialist nurses supported by experienced medical specialists.
- IT systems providing remote ‘**advice and guidance**’ to primary care and non-specialist colleagues are readily available but probably underutilised currently.
- Patients and caregivers should be directed to assured online resources and helplines set-up by specialist societies, organisations and patient groups to provide support during the pandemic (Table 1). It is important to have a consistent message that has been peer reviewed and quality assured that is available via a repository of resources to avoid mixed messages for patients and wasted time for clinical teams ‘reinventing the wheel’.

### Primary hyperparathyroidism

- In most instances, PHPT evolves slowly and many patients have had the disease for a long period (years) before diagnosis. Acute intervention is only rarely needed and work-up of patients referred with a new diagnosis of PHPT, including investigations to assess end-organ effects and localisation imaging should be postponed during the pandemic.
- Patients are advised to pay attention to potential **symptoms of hypercalcaemia** (e.g. anorexia, nausea, vomiting, bone and abdominal pain, osmotic symptoms and cognitive changes) and be aware of adequate fluid intake, in particular during the summer months. Patients should be advised to seek advice if they develop worsening and persistent symptoms of hypercalcaemia.
- Patients with PHPT should be made aware that they are susceptible to dehydration, particularly at times of intercurrent illness and hot temperatures. It is of paramount importance that patients keep well hydrated to avoid escalating hypercalcaemia and potential requirement for urgent care admission and renal insufficiency. Patients should be advised to keep well hydrated particularly if they have pyrexia.
- Routine serum calcium checks should not be made. Any management decisions should be based initially on changes in symptoms and clinical appearance.
- It is unlikely that definitive management of PHPT with PTX will be possible during the course of the pandemic. Patients should be reassured that in the short to medium term this is extremely unlikely to result in harm because end-organ effects such as nephrocalcinosis and reduced bone mineral density take many years to develop. In typical cases, surgery can be appropriately deferred until safe to proceed.
- **Cinacalcet** may be considered as a holding measure if there is symptomatic hypercalcaemia and no access to definitive PTX.

### Hypercalcaemia of malignancy

- Treatment of symptomatic hypercalcaemia is an important palliative consideration and there are well-established **guidelines** for this in view of its high prevalence in oncology. These guidelines mirror those for non-palliative care settings, as stated in the section on acute hypercalcemia of non-parathyroid origin.

### Hypoparathyroidism

- In patients with hypoparathyroidism, it is important to have an **uninterrupted supply of prescription medications** and this may require close liaison between endocrinologist and patients' primary care clinicians (https://parathyroiduk.org/news/coronavirus-advice-for-people-with-hypoparathyroidism/).
- Infections can impact on serum calcium/phosphate balance in hypoparathyroidism, pre-disposing to hypocalcaemia, so it is especially **important that patients are compliant** with their replacement medications to avoid the need for emergency admission. This can be reinforced by telephone or text reminders. Disease-specific emergency cards are helpful to highlight underlying diagnosis and planned treatments.
- Patients are advised to monitor symptoms closely, including those that may indicate hypocalcaemia (such as tingling sensations and muscle cramps) and to follow advice on self-management. If patients feel that their calcium is dropping, they are advised to **take additional calcium (500–1000 mg)** and consume calcium-rich foods or drinks.
- If symptoms do not improve with above, they are advised to seek urgent medical advice via local pathways, and this should trigger a blood test for calcium, albumin and creatinine with supporting advice from an endocrinology service.
- If doses of medications (particularly alfacalcidol or calcitriol) are changed, blood tests should be repeated within 1–2 weeks.
- In the event of a protracted **vomiting/ diarrhoea illness**, they should seek emergency medical advice as they are likely to require dose adjustments or even hospital admission.
- **Empirical dose changes of alfacalcidol or calcitriol should be avoided** in the absence of confirmatory blood testing.
- Patients should also be aware of **symptoms of hypercalcaemia**, which can also occur in the management of hypoparathyroidism. Persistent symptoms of hypercalcaemia despite appropriate hydration will also require medical review and further assessment.
- Patients on **parathyroid hormone replacement therapy** should continue their treatment as usual.

### Osteoporosis

- Detailed advice on management considerations and the impact of COVID-19 is outlined in Table 2.
- Particular attention should be made to patients receiving IV zoledronic acid, who we advocate delaying their next infusion by ~6 months or maybe longer, or according to progress of the pandemic. A standard letter is available on the Society for Endocrinology website (https://www.endocrinology.org/clinical-practice/covid-19-resources-for-managing-endocrine-conditions/).
- Patients established on **denosumab should continue their 6 monthly injections** and where appropriate measures to support self-injection or domiciliary administration should be explored.
- For denosumab-treated patients, pre-injection blood tests may be waived, and patients should be empirically treated with vitamin D supplementation (25,000–50,000 IU) around the time of each injection.
- Patients receiving **teriparatide or abaloparatide** should continue with their planned therapy. However, pauses from therapy for many weeks are very unlikely to blunt the long-term beneficial effect on fracture risk reduction.
- Arrangements for monthly administration of **romosozumab** by a health provider might need to be made or alternate therapies considered. Initiation of therapy with romosozumab should be delayed.
- Ideally, new patients should not be commenced on the above therapies during the acute COVID-19 response due to risk of confusion with regards to potential side effect profiles and symptoms of COVID-19.
- It is essential to have in place reliable databases and registries to ensure that patients do not get missed and have appropriate recall procedures when services can more safely be resumed.

**Table 2:** Summary of the approach to the management of osteoporosis during the COVID-19 pandemic.

General considerations	• **Next zoledronic acid infusion** can be delayed for at least 6–9 months
	• Patients who are established on **6-monthly denosumab (Prolia®)** injections should continue with their treatment without delay. The need for pre-injection blood test to check patients' serum vitamin D and calcium levels can be waived and empirical treatment with single dose of colecalciferol 25,000–50,000 IU can be considered for all patients.
	• Patients who are established on **teriparatide, abaloparatide or romosozumab** injection should continue with their treatment. Periods of discontinuation for many weeks are unlikely to blunt the long-term beneficial effects on fracture risk reduction, however.
	• No new patients should be started on **zoledronic acid infusion, teriparatide, romosozumab or abaloparatide** during the COVID-19 pandemic to avoid confusion due to the risk of developing mild ‘flu-like’ symptoms or developing shortness of breath, nausea and vomiting, fatigue and pain in the extremities with teriparatide.
	• **Alternative treatment such as continuing with oral bisphosphonate medication (if not contraindicated) should be considered.**
Educate	**All patients** should be educated on the importance of continuing with their calcium and vitamin D either by supplementation (if prescribed) or diet.
	• **All patients** should be educated on the importance of engaging in regular exercise. With the restrictions imposed by social distancing measures, patients should be educated on simple weight-bearing exercise routines that can be performed in the comfort their own home. Patients should be directed to reputable online resources for exercise such as the Royal Osteoporosis Society (https://theros.org.uk/information-and-support/living-with-osteoporosis/exercise-and-physical-activity-for-osteoporosis), the National Osteoporosis Foundation (https://www.nof.org/patients/treatment/exercisesafe-movement/osteoporosis-exercise-for-strong-bones/) and the international osteoporosis foundation (https://www.iofbonehealth.org/exercise).
	⚬ **All patients** should be educated on the importance of lifestyle measures such as healthy-balanced diet, maintaining a healthy body weight, avoiding smoking and minimising alcohol intake. During this period when self-isolation is highly imposed, many patients would be tempted to go back to old habits of smoking, sedentary lifestyle, unhealthy diet and increasing alcohol consumption. Patient education should focus on highlighting the importance of continuing with positive lifestyle measures. Patients can be directed to some useful online resources such as the UK National Centre for Smoking Cessation and Training (NCST) (https://www.ncsct.co.uk/usr/pub/smoking_and_bone_health.pdf), UK NHS website (https://www.nhs.uk/conditions/osteoporosis/), the Royal Osteoporosis Society (https://theros.org.uk/information-and-support/looking-after-your-bones/bone-health-checklist), the International Osteoporosis Foundation (https://www.iofbonehealth.org/nutrition), the National Osteoporosis Foundation (http://www.nof.org)
	• Patients who are either due to start or receive their **next zoledronic acid infusion** should be informed of the importance of delaying the treatment due to the potential side effects of mild ‘flu-like symptoms’ which could be mistaken as COVID-19 infection. Oral bisphosphonate should be considered as an interim treatment provided that there is no contra-indication for such treatment.
	• Patients should be informed and reassured about the long-acting nature of **zoledronic acid**. Patients should be reassured that their last infusion will continue to provide protection beyond 12 months (8) and therefore delaying their next infusion during the COVID-19 pandemic should not put them at a higher risk of sustaining fractures.Patients should be informed of the rapid onset and offset of action of **denosumab** treatment. Therefore, due to its quick ‘on and off’ effect, the administration schedule of every 6 months should not be interrupted or delayed.Patients should be reassured that continuing with their 6-monthly **denosumab** (Prolia®) injection should not put them at increased risk of contracting coronavirus.
	• If patients (or their family/carer) are willing **to self-administer** their denosumab injection, training on injection preparation and administration should be carried out either over the phone, by watching an online video or via video consultation.
	• Patients should be informed of the importance of continuing with their daily **teriparatide and abaloparatide** treatment. They should also be reassured that continuing with **teriparatide or abaloparatide** treatment should not put them at increased risk of contracting coronavirus.
Equip	Provide patients with tools and resources to support them in facilitating positive lifestyle changes including information and guide on exercise, healthy eating and smoking cessation. Patients could be directed to the following online resources: ⚬ https://theros.org.uk/information-and-support/living-with-osteoporosis/exercise-and-physical-activity-for-osteoporosis ⚬ https://www.ncsct.co.uk/usr/pub/smoking_and_bone_health.pdf ⚬ https://www.nhs.uk/conditions/osteoporosis/ ⚬ https://theros.org.uk/information-and-support/looking-after-your-bones/bone-health-checklist ⚬ https://www.iofbonehealth.org/nutrition ⚬ http://www.nof.org Provide patients with the details of dedicated service helpline or that of the patient support group for advice and support.
Engage	Osteoporosis is predominantly a disease affecting women over the age of 50 years and a significant proportion are over age of 70 and therefore at the high-risk category for contracting COVID-19. Family and carers should be involved in the care of patients and should be encouraged to support patients in observing stringent social distancing, regular handwashing and self-isolation when required.
	• To enable patients to continue with their **6-monthly denosumab** injection during the period of self-isolation, the possibility of self-administration by patient, or with the support of their family or carer, should be explored. Following this, patients (or their family/carer) can be guided on self-administration via video consultation to ensure safe preparation and administration techniques are followed on the day of Prolia injection.
	• **Engaging primary care clinicians.** Management plan and arrangement for patients to delay their **zoledronic acid treatment and teriparatide** should be clearly communicated to their primary care clinicians to ensure that other osteoporosis medication (e.g. calcium and/or vitamin D) prescriptions are not interrupted or delayed. For **denosumab** treatment (either through primary care, secondary care of self-administration by patients) it should be clearly communicated to their primary care clinicians to ensure that medication prescriptions are not interrupted or delayed. For patients who are at increased risk of contracting COVID-19 infection, where strict self-isolation is warranted, primary care engagement is never been more crucial and shared care arrangement should be discussed and encouraged. Shared care arrangement will enable primary care clinicians to continue prescribing and administering patients' denosumab injection without the need for patients to travel to the hospital.
	• **Engaging pharmaceutical companies**. Support that can be provided by pharmaceutical industry should be explored such as provision online, telephone support, as provision of home delivery of medication (e.g. teriparatide, denosumab), or homecare provision to assist patients on self-administration. Recently, in response to the COVID-19 pandemic, AMGEN has intensified its PROLONG support programme. PROLONG support programme is available for patient in Prolia injection and reminds patients of their next injection. The programme now offers dedicated nurse helpline which will support patient on self-administration.
	• **Engaging other osteoporosis expert nationally**. To ensure consistency in the delivery of osteoporosis care, clinicians specialising in osteoporosis management should be engaged at national level. Clinical guidelines and protocols with pragmatic approach to osteoporosis management should be shared. Sharing of innovative practice should also be facilitated through a national platform to avoid reinventing the wheel and facilitate early adoption in other centres. A good example of this is the initiative from the Society of Endocrinology (UK) where clinicians are sharing their local guideline and protocol for managing endocrine conditions including osteoporosis during the COVID-19 pandemic (https://www.endocrinology.org/clinical-practice/covid-19-resources-for-managing-endocrine-conditions/)
	• **Engaging patient support/advocacy groups**. A key stakeholder in osteoporosis management is the patient support/advocacy group. Osteoporosis patient groups are very active in most Westernised country including the UK. Informing them of the approach being adopted locally or regionally on the management of patients with osteoporosis during COVID-19 outbreak will enable them to provide appropriate support to their members and the public.
Empower	• Well-informed and well-equipped patients are likely to be empowered to facilitate self-management.
	• Provide patients with the details of dedicated service helpline or that of the patient support group for advice and support.

## In patients with calcium metabolic disorders and osteoporosis, who still needs to be seen face-to-face?

- Patients with calcium metabolic disorders who are biochemically stable, requiring follow up intervals greater than 3 monthly, can generally be managed remotely as mentioned above. Use phlebotomy services, according to clinical need, that are located away from acute hospitals with minimal footfall and risk of infection.
- Those with calcium homeostasis problems that are less stable, measured by requiring follow up intervals less than 3 monthly, should again receive remote blood tests, and these should be acted upon promptly by contact to patient and primary care clinician by phone, text, e-mail and corroborated by formal letter documentation.
- Only in exceptional clinical circumstances, or in those requiring **emergency assessment**, addressed above, will patients with problems of calcium homeostasis require face-to-face review.
- Patients with osteoporosis being treated with **denosumab or romosozumab** may require face-to-face interactions (addressed above) for drug administration if they are not self-administering.

## Which online resources are available for patients with calcium disorders and osteoporosis?

See Table 1.

## What might be the longer-term consequence for service provision?

- The aftermath of the pandemic will not be characterised by a return to business as usual. There will be a requirement for addressing large numbers with postponed care and need for clinical assessments. It is essential to have in place reliable databases and registries to ensure that patients are not missed and have appropriate recall procedures for administration of drug therapies when services can be resumed more safely.
- When effective vaccines become available, patients with calcium metabolic disorders and osteoporosis should access such.
- The collective response to COVID-19 has served as a catalyst to innovation in our management of mineral and metabolic bone diseases.
- During the acute phase of the pandemic we have postponed non-urgent elective consultations, tests and therapeutic interventions.
- Reinstitution of elements of services should be guided by risk assessment of disease categories and individual patient scenarios.
- Patients with less stable disorders of calcium balance should be prioritised when re-implementing services.
- By **empowering patients** to take on more control of their disease management and supporting with remote appointments and digital health solutions we will be able to build more resilient systems.
- Patient centred management, **coordination between primary and secondary care** and building stronger national and international ties are lasting legacies of the pandemic.
- The crisis has highlighted that we share common challenges and vulnerabilities and that the solutions lie in sharing knowledge and resources to enable a collective response.

## Disclaimer

Due to the emerging nature of the COVID-19 crisis, this document is not based on extensive systematic review or meta-analysis, but on rapid expert consensus. The document should be considered as guidance only; it is not intended to determine an absolute standard of medical care. Healthcare staff need to consider individual circumstances when devising the management plan for a specific patient.

## Declaration of interest

The authors declare that there is no conflict of interest that could be perceived as prejudicing the impartiality of this guidance.

## Funding

This research did not receive any specific grant from any funding agency in the public, commercial or not-for-profit sector.
